# Seasonal Changes in Color Preferences Are Linked to Variations in Environmental Colors: A Longitudinal Study of Fall

**DOI:** 10.1177/2041669517742177

**Published:** 2017-12-04

**Authors:** Karen B. Schloss, Isobel A. Heck

**Affiliations:** Department of Psychology and Wisconsin Institute for Discovery, University of Wisconsin–Madison, WI, USA; Department of Human Development, Cornell University, Ithaca, NY, USA

**Keywords:** color, cognition, aesthetic preferences, individual differences

## Abstract

People form associations between colors and entities, which influence their evaluations of the world. These evaluations are dynamic, as specific associations become more or less active in people’s minds over time. We investigated how evaluations of colors (color preferences) changed over the course of fall, as color-associated fall entities became more prevalent in the environment. Participants judged their preferences for the same set of colors during nine testing sessions over 11 weeks during fall. We categorized the colors as Leaf and Non-Leaf Colors by matching them to leaves collected during the same period. Changes in preferences for Leaf Colors followed a quadratic pattern, peaking around when the leaves were most colorful and declining as winter approached. Preferences for Non-Leaf Colors did not significantly change. Individual differences in these changes could be explained by preferences for seasonal entities, as predicted by the differential activation hypothesis within the Ecological Valence Theory. The more a given individual liked fall-associated entities, the more their preference for Leaf Colors increased during fall. No analogous relations existed with winter-associated entities or Non-Leaf Colors. These results demonstrate the importance of studying temporal and individual differences for understanding preferences.

As people interact with the world, they form associations between colors and entities. They learn that ripe bananas are yellow, clear skies are blue, and fresh leaves are green. They also form abstract color-entity associations: The University of Wisconsin–Madison and Cornell University are associated with reds, Thanksgiving and Halloween are associated with oranges, and the U.S. Democratic Party and Facebook are associated with blues. Evidence suggests that color-entity associations influence people’s responses to colors in a variety of domains. They influence people’s percepts of how colors appear (Hansen, Olkkonen, Walter, & Gegenfurtner, 2006), people’s ability to interpret the meaning of colors in data visualizations (Lin, Fortuna, Kulkarni, Stone, & Heer, 2013), and people’s aesthetic preferences for colors (Palmer & Schloss, 2010).

Understanding how color-entity associations influence people’s evaluations and interpretations of the world is a complex problem for two main reasons. First, these mappings are not one-to-one: The same color can be associated with many entities and many colors can be associated with the same entity (Elliot & Maier, 2012; Humphrey, 1976; Lin et al., 2013; Setlur & Stone, 2016). For example, one particular shade of red might be simultaneously associated with apples, roses, the U.S. Republican Party, and the University of Wisconsin–Madison. And, many different shades of reds, as well as yellows and greens, may all be associated with apples. Second, different entities that are associated with colors are more or less relevant to an observer at a given moment of time (Schloss, Nelson, Parker, Heck, & Palmer, 2017; Schloss & Palmer, 2014; Strauss, Schloss, & Palmer, 2013). For example, in the United States, the association between red and the U.S. Republican Party may be particularly relevant on days with important elections, but that association may be much less relevant while apple picking in October. Moreover, although the association between red and the U.S. Republican Party may be relevant to some people, this same association may be irrelevant or unknown to others who are unaffected by U.S. politics.

Evidence suggests that changes in which entities are active in people’s minds can lead to variations in color preferences. In a controlled laboratory study, priming observers to think about positive or negative entities associated with particular colors resulted in changes in preferences for those colors (Strauss et al., 2013). For example, participants’ preferences for red significantly increased after being primed with images of positive red entities (e.g., strawberries and roses) and slightly decreased after being primed with images of negative red entities (e.g., blood and lesions). Systematic changes in color preferences also occur with variations outside of the laboratory, such as variations in activation of political affiliation over political voting cycles (Schloss & Palmer, 2014) and variations in activation of seasonal entities over seasonal changes in the environment (Schloss et al., 2017).

In this study, we aimed to further understand seasonal variations in color preferences by investigating (a) whether changes in color preferences during fall were linked to changes in environmental colors, namely changing leaves, and (b) whether individual differences in seasonal color preference changes were linked to individual differences in preferences for seasonal entities.

The notion that changes in the activation of color-associated entities results in changes in color preferences can be explained within the Ecological Valence Theory (EVT; Palmer & Schloss, 2010). The EVT proposes that an individual’s preference for a particular color is determined by their combined valence (liking or disliking) of all entities associated with that color. Colors that are especially liked (e.g., saturated blue) tend to be associated with mostly positive entities (e.g., clear sky, clean water), whereas colors that are especially disliked (e.g., dark yellow) tend to be associated with more negative entities (e.g., biological waste, rotting food), on average. The EVT further specifies that color preferences act as an adaptive steering function that guides organisms to approach entities that will lead to positive outcomes and avoid entities that will lead to negative outcomes. Expanding on the initial framework of the EVT, Schloss and Palmer (2017) describe three hypotheses for why color preferences should change over time and differ between individuals. The hypothesis most relevant here is the *differential activation hypothesis.*

The differential activation hypothesis specifies that preferences for particular colors tend to change depending on the degree to which entities associated with those colors are active in an observer’s mind (Schloss & Palmer, 2017). We assert that color preferences *tend* to change because the direction and magnitude of this change will depend on the valence of the activated entity, relative to all entities associated with the color. Say entity *e* is associated with color *c* and the degree to which entity *e* is active in the mind of observers varies over time. And, say that an observer *s_1_* likes entity *e* more than their mean liking for all entities associated with color *c*, observer *s_2_* likes entity *e* less than their mean, and observer *s*_3_ likes entity *e* equally well as their mean. If all else is equal, the differential activation hypothesis predicts that increasing the activation in entity *e* will cause an increase in preference for color *c* for *s_1_*, a decrease for *s_2_*, and no change for *s_3_*. This hypothesis implies that in calculating an individual’s color preferences at a given moment, the mind gives greater weight to color-associated entities that are more relevant to the individual at that time. If color preferences help to steer individuals toward positive outcomes and away from negative ones as the EVT suggests, then it is beneficial for the system to titrate the weight given to different entities that are more or less relevant, depending on the context.

Although the differential activation hypothesis is contingent on the valence of entities associated with a color, it is distinct from the *differential valence hypothesis.* The differential valence hypothesis states that preferences for a particular color will tend to change if preferences for entities associated with that color change over time (Schloss & Palmer, 2017). Further, it states that individuals will differ in their preference for a color at a given moment in time if they have different preferences for the same entities associated with that color. The difference between these hypotheses that is most relevant here concerns the predictions for why color preferences *change* over time. Whereas the differential valence hypothesis predicts preference for a color changes because preferences for entities associated with that color change, the differential activation hypothesis predicts preference for a color changes because the extent to which entities associated with that color are activated in the mind changes. As described in the previous paragraph, the effect of changing activation of a particular entity depends on the individual’s preference for that entity.

Schloss et al. (2017) provided initial evidence that the differential activation hypothesis, and not the differential valence hypothesis, accounted for seasonal variations in average color preferences. Participants judged their preferences for the same set of colors during fall, winter, spring, and summer in the Northeastern United States, a region in which there are dramatic seasonal changes in weather and environmental colors. After making their last set of color preference judgments, participants rated how strongly they associated each color with each season. The seasonal differences in color preferences were between fall and the average of the three other seasons. Participants liked dark-warm colors, which were strongly associated with fall, more during the fall testing session than during the other three testing sessions. And they liked light-warm, light-cool, and dark-cool colors less during fall than during the other three testing sessions. Schloss et al. (2017) noted that from their data, it was unknown whether the difference between fall versus the other seasons was partially due to an order effect, given that fall was the first testing session. We address that issue in this study.

With data from different participants, Schloss et al. (2017) evaluated whether these seasonal changes in color preferences could be predicted by (a) seasonal changes in the activation of entities associated with the colors tested (differential activation hypothesis), (b) seasonal changes in the valences of entities associated with the colors tested (differential valence hypothesis), or (c) both. They tested these hypotheses using a variant of Palmer and Schloss’s (2010) Weighted Affective Valence Estimate (WAVE) procedure to calculate three variants of seasonal WAVEs. The WAVE for a given color is a single number that represents how much people like the entities associated with that color, on average (weighted by the degree to which the color associated with each entity matches that color). The three types of seasonal WAVEs modified the original WAVE equation to code for (a) seasonal activation of seasonal entities, (b) seasonal valences of seasonal entities, and (c) both (see Schloss et al., 2017 for methodological details and equations). Differences in color preferences (fall vs. other seasons) were significantly predicted by WAVEs that coded for seasonal differences in activation (*r* = .51) but not by WAVEs that coded for seasonal differences in valence (*r* = .29). WAVEs coding for seasonal variations in activation and valence fit the data no better than WAVEs coding for seasonal activation alone (*r* = .52). The results suggest that seasonal changes in color preferences are due to differential activation and not differential valences over time.

In this study, we address open questions that were motivated by Schloss et al.’s (2017) initial study on seasonal variations in color preferences. First, we examined whether changes in color preferences during fall were linked to changes in environmental colors (i.e., changing leaves). Second, we tested whether individual differences in how color preferences changed over fall could be predicted by individual differences in preferences for seasonal entities. The differential activation hypothesis predicts that as fall entities became more prevalent during fall, and thereby more active in people’s minds, people’s preferences for colors associated with fall entities would increase to the extent that they like fall entities. Third, we examined whether an order effect was responsible for the previously observed preference changes in fall relative to the other seasons.

We addressed these questions by conducting a within-subject longitudinal study, spanning nine testing sessions over 11 weeks around Fall 2014. Participants rated their preferences for each of the Berkeley Color Project 37 (BCP-37) colors (Palmer & Schloss, 2010; Schloss, Strauss, & Palmer, 2013) during each testing session. This timescale gave us a finer resolution than that given in the study by Schloss et al. (2017), in which participants only judged their preferences for those colors during one session per season. At the end of our study, the same individuals judged their preferences for fall- and winter-associated entities. During the study, we provided participants with no explanation for why we tested their color preferences repeatedly each week. Throughout the study, we documented changes in the local environmental colors by taking photographs from the same locations each week. We also determined which of the BCP-37 colors matched the colors of fallen autumn leaves. Reasoning that autumn leaves are fall-associated entities that provide strong and prevalent visual cues to seasonal changes, we focused our analyses on changes in individuals’ preferences for “Leaf Colors” versus “Non-Leaf Colors.”

## Methods

### Participants

Thirty-two participants were recruited for the study but the analyses were conducted on 22 participants (15 females, *M_age_* = 21 years).^1^ The criteria for including participants were determined a priori: (a) having typical color vision determined using the HRR Pseudoisochromatic Plates (Hardy, Rand, Rittler, Neitz, & Bailey, 2002; one participant excluded); (b) completing all nine testing sessions (four participants excluded); and (c) having stable color preferences within the first testing session (five participants excluded). Stable color preferences were operationalized as a within-subject correlation between color preference ratings during Block 1 and Block 2 of the first testing session that was >.70 (Schloss et al., 2017; Strauss et al., 2013). All participants lived on or around Brown University’s campus in Providence, RI. All gave informed consent, and the IRB of Brown University approved the experimental protocol.

### Design, Displays, and Procedure

All participants completed two kinds of tasks: color preference ratings and entity preference ratings. Displays for both tasks were generated using Presentation software (www.neurobs.com) and were shown using an Asus ProArt PA246Q monitor (1920 pixel × 1200 pixel resolution, 51.9 cm wide × 32.5 cm tall). Participants completed the tasks in a dark, enclosed booth. They viewed the monitor from a distance of approximately 60 cm. Over the course of testing, we documented changes in environmental colors by taking photographs each week. We also collected autumn leaves from the ground to determine which BCP-37 colors best corresponded to the Leaf Colors (described later).

#### Color preference ratings

**Figure 1.**
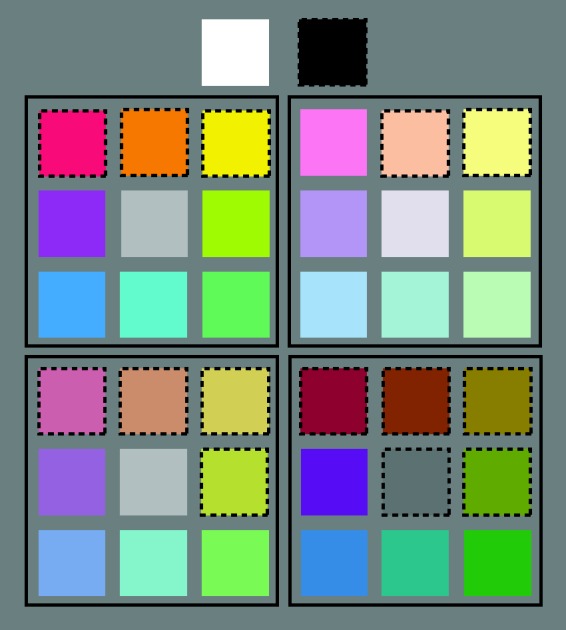
The BCP-37 colors. Each of the eight hues (red, orange, yellow, chartreuse, green, cyan, blue, and purple) were sampled at four saturation/lightness levels: saturated (top-left quadrant), light (top-right quadrant), muted (bottom-left quadrant), and dark (bottom-right quadrant). Each quadrant also includes a shade of gray whose luminance is similar to the average luminance of the colors within each quadrant. The grays depicted in the saturated and muted quadrants are identical. Black and white are depicted at the top of the figure. The dashed lines around some of the colors indicate that they were considered “Leaf Colors” (see text for details).

Participants judged their preferences for the BCP-37 colors (Palmer & Schloss, 2010; Schloss et al., 2013; Figure 1). The color set includes eight hues (red, orange, yellow, chartreuse, green, cyan, blue, and purple) sampled at four saturation/lightness levels (saturated, light, muted, and dark) as well as five achromatic colors (black, dark gray, medium gray, light gray, and white). The colors were sampled in Munsell space and translated to CIE 1931 xyY coordinates using the Munsell Renotation Table (Wyszecki & Stiles, 1967; see Appendix Table A1 for CIE 1931 xyY coordinates). To ensure accurate presentation of the colors, we characterized the monitor using a Konica Minolta CS-200 Chroma Meter and then measured the resulting colors. The measured colors had CIE x and y values that deviated by <.01 from the target values in Table A1 and Y (luminance) values that deviated by less than 2 cd/m^2^ from the target colors. The background approximated CIE Illuminant C (CIE x = 0.312, y = 0.318, Y = 19.26).

During the experiment, the colors were presented as small squares (100 pixel × 100 pixel; 2.58° × 2.59°) centered on the screen. A 400-pixel-long (10.3°) response scale appeared below the color square with the left endpoint labeled as *not at all*, the right endpoint labeled as *very much*, and a central marker denoting neutral preference. The colors were presented one at a time in a blocked, randomized design (two blocks), such that participants rated their preference for all of the colors once, in a random order, before judging them a second time in a new random order.^2^ Participants rated how much they liked each color by sliding a cursor along the response scale and clicking to record their response. The responses were scaled to range from −100 to +100. Trials were separated by a 500-ms intertrial interval. Before starting the task, participants were shown the full set of colors and asked to complete an anchoring procedure (Palmer, Schloss, & Sammartino, 2013) so they understood what liking *not at all* and liking *very much* meant to them in the context of the color set.

**Figure 2.**
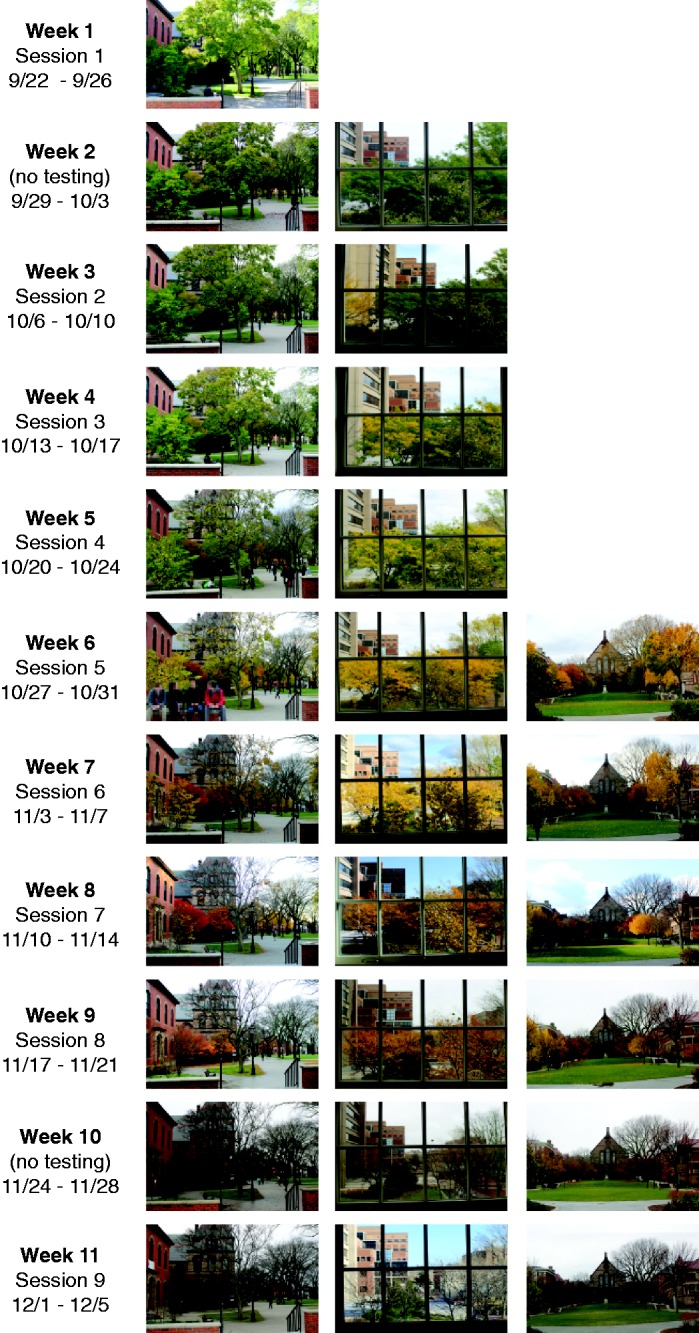
Photographs were taken from three locations on the Brown University campus during each week of the testing period. Photographs were taken from locations participants would pass on their way to testing sessions, including from the window of the laboratory in which they were tested (middle column). It is apparent from examination of the photographs shown in the figure that the peak of autumn leaf change occurred around Week 7.

Participants reported their color preferences using this procedure on nine separate testing days over 11 weeks (see Figure 2). There were no testing sessions during Week 2 or Week 10. For the most part, each participant had a regular testing day and time each week (e.g., Wednesdays at 11 a.m.), with only slight deviations if necessary to accommodate participants’ unavoidable scheduling conflicts. Participants were given no explanation for this repeated testing until after the entire study was complete.

#### Entity preference ratings

At the end of the last session (Session 9, Week 11), after participants made their last color preference judgments, they were asked to judge how much they liked seasonal entities. The entity descriptions were presented on the monitor as black text on a white background. First, participants rated their preference for each season (“fall,” “winter,” “spring,” and “summer”) in a random order. Next, they rated their preference for the fall- and winter-associated entities from Schloss et al. (2017), presented in a random order (see Appendix Tables A2 and A3). Ratings were made using the same continuous line-mark slider scale from the color preference task, with the endpoints labeled as *not at all* and *very much*. Trials were separated by a 500-ms intertrial interval. Before beginning, participants were presented with a set of entity descriptions that varied broadly in valence so they could anchor what liking *not at all* and liking *very much* meant for them in the context of this set of entities.

#### Documenting changes in environmental colors

To track the time course of changes in environmental colors over the course of the study, we took photographs from the same locations outside the lab each week (on Brown University’s campus), as shown in Figure 2. We also added additional locations as the study progressed to capture the changes where they were most prevalent, including a view through the lab window outside of the enclosed, dark testing booths. It is apparent that the peak of the autumn leaf colors was around Week 7.

#### Matching BCP-37 colors to environment colors

**Figure 3.**
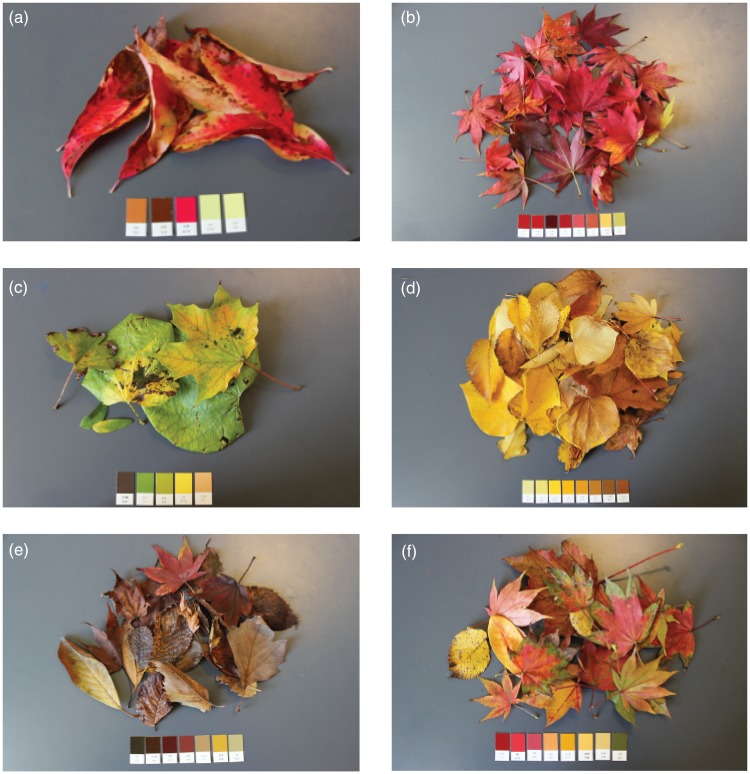
Images of the leaves collected on the Brown University campus during autumn and corresponding Munsell chips that matched them. The Munsell notation (Hue Value/Chroma) for each set of Munsell chips (left to right) within each panel were: (a) 5YR 5/6, 5YR 3/4, 2.5R 4/14, 10Y 8.5/6, 7.5Y 9/6; (b) 7.5R 3/10, 7.5R 3/12, 7.5R 2/6, 5R 3/10, 5R 4/8, 10R 4/6, 10YR 7/8, 7.5Y 6/6; (c) 7.5 YR 3/2, 5GY 5/6, 10Y 6/8, 5Y 8/10, 7.5YR 7/6; (d) 5Y 8/6, 5Y 8/8, 2.5Y 8/12, 10YR 7/12, 10YR 6/10, 10YR 5/8 10YR 4/6, 7.5YR 4/6; (e) 5Y 2/2, 7.5YR 2/4, 10R 2/4, 10R 3/4, 10YR 5/4, 2.5Y 6/8, 5Y 6/4; (f) 7.5R 3/10, 5R 4/12, 5R 4/8, 5YR 6/8, 10YR 6/10, 10YR 7/8, 2.5Y 7/6, 10Y 4/6.

To compare the colors that we observed in the environment to the colors that participants rated, we matched autumn leaf colors that had fallen from the trees around Week 6 to the closest BCP-37 colors. Doing so allowed us to know which subset of the BCP-37 colors to examine to determine if changes in participants’ ratings were linked to changes in environmental colors. To match the colors, we completed the following procedure: (a) We collected samples of autumn leaves that had fallen to the ground and brought them back to the lab; (b) we determined which Munsell color chips best matched the leaf colors (see Figure 3) while viewing them under sunlight that entered the space through large windows around noon, (c) we converted the matched Munsell coordinates to CIE xyY coordinates using the Munsell Renotation Table (Wyszecki & Stiles, 1967) and then converted those to CIELAB coordinates (using the CIE xyY coordinates of the background from the color preference task as the white point), and (d) we determined which of the BCP-37 colors best matched each leaf color sample by calculating the Euclidian distance (Δ*E*) between each leaf color sample and all BCP-37 colors in CIELAB space and selecting the BCP-37 color with the smallest Δ*E*.

We then verified these Δ*E* matches by having an independent set of five observers view each leaf color sample along with the full set of BCP-37 colors. On each trial, the observers saw one leaf color sample (as a square patch on the screen) and all BCP-37 colors (as displayed in Figure 1, without the outlines). They were asked to click on the BCP-37 color that best matched the presented leaf color sample. Their responses included no colors that were not already in the set of Δ*E* matches, and the set of Δ*E* matches did not include any colors that were not in the human matches. Therefore, we considered the set of Δ*E* matches our BCP-37 Leaf Colors, subsequently referred to as “Leaf Colors,” and the remaining BCP-37 colors the “Non-Leaf Colors.” Figure 1 denotes Leaf Colors with dashed outlines and Non-Leaf Colors with no outlines.

## Results and Discussion

### Changes in Average Preferences for Leaf Colors Versus Non-Leaf Colors

**Figure 4.**
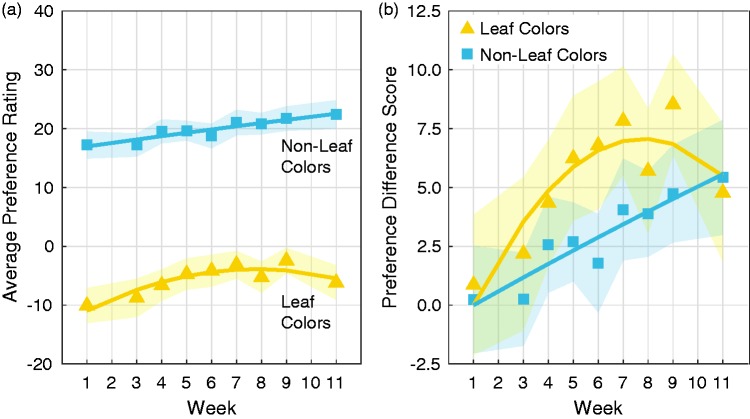
Average preferences for Leaf Colors (yellow triangles) and Non-Leaf Colors (blue squares) as a function of Week (a) and preference difference scores for these same color sets after adjusting for the Week 1 baseline (b). Curves represent best-fit quadratic functions through the data. Error regions in each graph represent ±1 SEM after accounting for overall differences between participants (i.e., overall difference in preferences averaged over all colors; Cousineau, 2005).

Figure 4(a) shows average preferences for Leaf Colors versus Non-Leaf Colors as a function of week of the testing period. A repeated measures analysis of variance (2 Color Sets (Leaf vs. Non-Leaf) × 9 Testing Sessions) indicated that the Non-Leaf Colors were generally more preferred than the Leaf Colors, *F*(1, 21) = 42.91, *p* < .001, $\eta_{\text{p}}^{2}$ = .67. This is expected because the Non-Leaf Color set includes the blues and blue-greens that people generally like and the Leaf Color set includes the dark yellows and yellow-greens that people generally dislike (Granger, 1955; Guilford & Smith, 1959; Hurlbert & Ling, 2007; McManus, Jones, & Cottrell, 1981; Palmer & Schloss, 2010; Taylor & Franklin, 2012).

In testing for changes in color preference over time, we found an interaction between Color Set and a quadratic contrast over Testing Session, *F*(1, 21) = 9.33, *p* = .006, $\eta_{\text{p}}^{2}$ = .31. Follow-up tests within each level of Color Set indicated that there was a significant quadratic contrast over Testing Session for Leaf Colors, *F*(1, 21) = 13.6 *p* = .001, $\eta_{\text{p}}^{2}$ = .39, but not for Non-Leaf Colors (*F* < 1). This pattern is more apparent in Figure 4(b), which shows the average preferences for Leaf Colors and Non-Leaf Colors after subtracting the estimated Session 1 baseline for each color set (calculated from the quadratic fits). Although it appears that there was a linear increase in preferences for Non-Leaf Colors, the linear contrast was not significant, *F*(1, 21) = 3.84, *p* = .063, $\eta_{\text{p}}^{2}$ = .16. The time course for changes in preferences for Leaf Colors resembled the time course for changes in environmental colors (Figure 2). The peak of the quadratic function for Leaf Colors was at Week 7.8 (Figure 4), which was also around the time at which the leaves were most colorful (Figure 2). As the season turned from summer to fall and the leaves turned from greens to yellows and oranges, preferences for Leaf Colors increased. As winter approached and the leaves fell, preferences for Leaf Colors decreased.

For the most part, the average data points for Leaf Colors follow a smooth quadratic function (Figure 4), but there is a clear dip at Week 8 (November 10 to November 14). It is as though the curve starts to decline at Week 8 but then jumps back up at Week 9, only to continue declining by Week 11. We anticipated there might be a surge in preferences for Leaf Colors around Week 9 because the Thanksgiving holiday (and Thanksgiving break from classes) was at the end of Week 9. It is possible that preferences for Leaf Colors were on the decline around Week 8 but that decrease was interrupted by the activation of Thanksgiving in the participants’ minds, along with its related activities, decorations and events. Thanksgiving is associated with similar colors as the Leaf Colors and is associated with relatively positive valence, on average (Schloss et al., 2017). Following the differential activation hypothesis, increasing activation for Thanksgiving should increase preference for its associated colors. Indeed, the increase in Leaf Color preference from Week 8 to Week 9 was significant, *F*(1, 21) = 4.53, *p* = .045, $\eta_{\text{p}}^{2}$ = .18, as was the decrease from Week 9 to Week 11, *F*(1, 21) = 4.65, *p* = .043, $\eta_{\text{p}}^{2}$ = .18 (there was no testing during Week 10 because of the Thanksgiving holiday).

### Individual Differences and the Differential Activation Hypothesis

**Figure 5.**
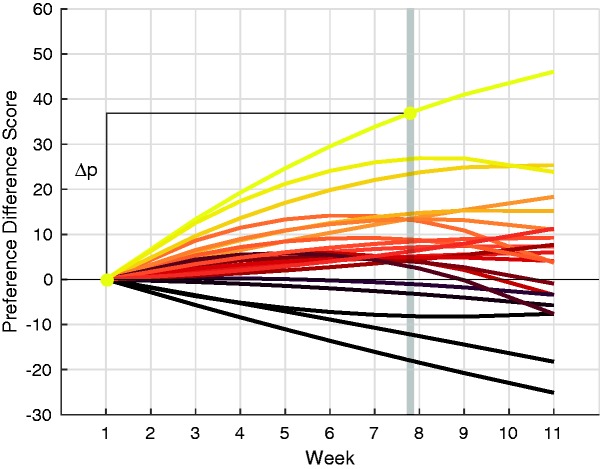
The best-fit quadratic function for each individual’s change in preference for Leaf Colors as a function of week in the study. Each participant’s preference at week 7.8 (the peak Leaf Color preference across participants depicted by the gray stripe) was used to calculate the magnitude of each individual’s change in Leaf Color preference from Week 1 (Δp). The color assigned to each participant corresponds to their rank order in the distribution of Δp values for Leaf Colors such that each participant has a unique color (the color is not mapped onto the quantity of Δp).

We next tested whether individual participants’ changes in color preferences during fall depended on how much they personally liked fall-associated entities, as predicted by the differential activation hypothesis. To do so, we first quantified the change in preference for each individual, which we call Δp. For each participant, we fit a quadratic function to their preferences for Leaf Colors. Figure 5 shows each individual’s quadratic function after subtracting that participant’s estimated Session 1 baseline (calculated from the quadratic fits) from their Leaf Color preferences during each week (analogous to average data in Figure 4(b)). We then used the quadratic function to calculate each individual’s estimated preference for Leaf Colors at Week 7.8, which was the time at which the average Leaf Color preferences peaked, as described earlier. Finally, we calculated Δp as the difference in the individual’s estimated preference at Week 7.8 from their estimated preference at Week 1. This is equivalent to the magnitude of the preference difference score in Figure 5 after subtracting out the Week 1 baseline. The individuals’ curves in Figure 5 are colored by sorting their Δp values for Leaf Colors from high to low and then assigning each participant a unique color according to their rank order.

**Figure 6.**
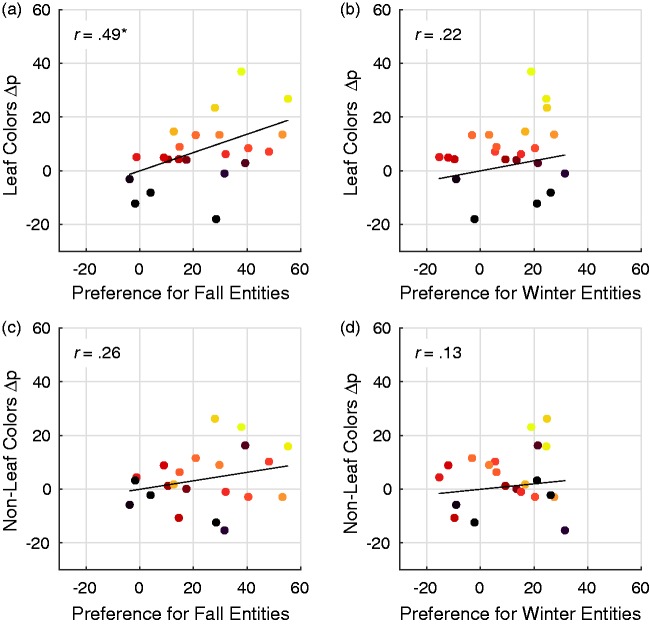
The relation between preference for fall- and winter-associated entities and the magnitude change in individual participants’ preferences for Leaf and Non-Leaf Colors. There was a significant relation between preference for fall entities and individuals’ preference change for Leaf Colors (Δp) (a), but not between winter-associated entities and Leaf Colors (b), fall-associated entities and Non-Leaf Colors (c), and winter-associated entities and Non-Leaf Colors (d). Individual data points represent each of the 22 participants, and the color assigned to each participant is the same as their color in Figure 5.

As shown in Figure 6(a), there was a significant positive correlation between individuals’ Δps for Leaf Colors and their preferences for entities associated with fall (averaged over all entities), *r*(20) = .49, *p* = .021. Participants who had a greater preference for fall entities showed greater increases in preferences for Leaf Colors during fall. Figure 6(b) shows that there was no corresponding relation between Leaf Color Δps and preference for winter entities, *r*(20) = .22, *p* = .332. This result suggests that the relation between Leaf Colors and fall entities (Figure 6(a)) was specific to fall, and not due to a relation between Leaf Color Δps and preference for seasonal entities more generally. To ensure that the relation in Figure 6(a) was not due to a general relation between changes in color preferences and preferences for fall entities, we calculated Δp for Non-Leaf Colors using the method described earlier. Non-Leaf Color Δps were not related to preferences for fall entities, *r*(20) = .26, *p* = .246 (Figure 6(c)), or to preferences for winter entities, *r*(20) = .13, *p* = .551 (Figure 6(d)).^3^ These results suggest that the relation between Leaf Color Δps and preferences for fall entities was indeed due to a meaningful relation between Leaf Colors and fall entities.

We also tested whether there were similar relations between Δp for Leaf Colors and preferences for the season in general (i.e., “fall”). However, the relation between Δp and preference ratings for “fall” was not significant, *r*(20) = .18, *p* = .426. Why might Leaf Color Δps relate to average preference for fall entities but not to the umbrella term to which these entities belong? One possibility is that individuals’ preference ratings for “fall” are single data points, resulting in a noisier measure than average preference ratings for all 43 entities associated with fall. Another possibility is that the term *“*fall” is too abstract to elicit the affective responses in observers that are elicited by naming particular entities associated with fall (e.g., pumpkin spice lattes, hay rides, Halloween). Future research will be necessary to test these hypotheses.

## General Discussion

We investigated how color preferences vary over the course of fall, why such changes might occur, and why they differ among individuals. We assessed the same participants’ color preferences during nine sessions over an 11-week period (late September 2014 to early December 2014). At the beginning of the study, the colors in the environment were as they were in summer—the trees were lush green and the flowers were colorful. By the end, the colors in the environment resembled winter—the trees were bare brown and the flowers were gone. In the interim, the leaves on the trees turned shades of reds, oranges, and yellows. As the colors changed, fall-specific objects, entities and events such as pumpkin spice lattes, hayrides, cooler weather, apple picking trips, Halloween, and Thanksgiving were increasingly prevalent in the region where we conducted our study. Over this time period, we tracked our participants’ preferences for Leaf Colors (i.e., colors that corresponded to the colors of fallen autumn leaves near our testing location) and Non-Leaf Colors (i.e., the remaining colors in our color set). Partitioning the colors in this way allowed us to test whether seasonal changes in color preferences correspond to seasonal changes in environmental colors, which are associated with seasonally specific entities.

We tested the differential activation hypothesis, which predicted that preferences for fall-associated colors would increase as fall entities became more active in observers’ minds, so long as those entities were relatively liked. In line with this prediction, average preferences for Leaf Colors increased as fall progressed and then decreased as fall ended and winter approached. There was also a small peak in the function around the Thanksgiving holiday, when fall-associated entities would have been especially activated. There were no corresponding changes in preferences for Non-Leaf Colors.

The quadratic pattern of average preferences for Leaf Colors also suggests that the previously reported seasonal changes in color preferences were not due to an order effect. Schloss et al. (2017) found that participants liked dark-warm colors (which are included in our Leaf Colors) more during fall (first testing session) than during the other three seasons (second to fourth testing sessions). Here, Leaf Colors were least-preferred during our first testing session and preferences increased toward the peak of fall, which is around when Schloss et al. (2017) had collected their data a few years prior. Therefore, people like fall-associated colors more during the peak of fall, regardless of when testing begins.

We also found extensive individual differences in the degree to which preferences for Leaf Colors changed over fall, which were predicted by how much individuals liked color-associated fall entities. Yet, despite this individual variation, the majority of participants reported liking fall entities and as such showed increases in preferences for Leaf Colors over the testing sessions. If our participants’ preferences for fall entities had more uniformly spanned the range from negative to positive, we would not have observed a systematic change in the average data; the changes at the individual level would have cancelled out. This point emphasizes the need for considering individual differences when studying changes in preferences, and understanding the individualized circumstances that give rise to those changes. Without doing so, diverse and systematic individual changes in preferences could be missed and wrongly interpreted as absent.

Our interpretation of the present results is that changes in activation of seasonal entities caused changes in preferences for associated colors, rather than the reverse causal direction. Thus, we propose that individual differences in liking of fall-associated entities caused individual differences in the direction and magnitude of change in preference for Leaf Colors. Our present data do not explain why there are individual differences in preferences for fall entities, but these differences are likely due to personal experiences. For example, imagine two participants, *s_1_* and *s_2_,* took trips to the same farm and partook in the same activities (e.g., apple picking, hay rides, and pumpkin carving), but their experiences had different valences. Participant *s_1_* had fun with friends (positive valence), whereas participant *s_2_* broke up with their significant other (negative valence). The different valences of these two experiences would cause the related activities (apple picking, hay rides, and pumpkin carving) to have positive valences for *s_1_* and negative valences for *s_2_.*

However, it is worth considering the opposite causal direction, which proposes that increased preferences for fall colors causes increased preferences for fall entities. If that were the case, it would still be necessary to explain the increased preferences for fall colors. One possible account is mere exposure (Zajonc, 1968, 2001); people may have come to like Leaf Colors more during fall because they saw those colors more in fall. To evaluate this possibility, it is relevant to consider the valence of the stimuli (in our case colors) at the start of the study. Classic mere exposure studies that demonstrated an increase in liking for stimuli after more exposure to them typically used stimuli that were neutral at the start of the study (Zajonc, 1968, 2001). However, Brickman, Redfield, Harrison, and Crandall (1972) found that preferences for initially disliked stimuli may actually decrease, rather than increase, with exposure to them. We found that Leaf Colors were initially disliked, yet preference for those colors increased over time (Figure 4(a)). This pattern is the opposite of the exposure pattern predicted by Brickman et al. (1972), which challenges the account that the observed changes in color preferences were due to mere exposure. To fully test the effects of exposure on changes in color preferences, future work might carefully control exposure in a laboratory setting or collect viewing statistics in a natural setting to test the extent to which color preferences are correlated with the colors that individual people experience.

In conclusion, the results of this study support the notion that people’s associations between colors and entities influence the way they evaluate the world. Some aspects of these associations are shared across individuals (e.g., all participants in our study walked past the autumn leaves on campus and had a holiday from classes due to Thanksgiving), but other aspects are highly individualized, including people’s affective responses to these color-associated entities. Further, the relevance of particular color-entity associations to an individual’s goals and desires can change over time (e.g., pumpkin spice lattes might be highly sought in fall but are irrelevant during other seasons because they are unavailable in coffee shops). These individual and temporal variations in color-related experiences contribute to systematic temporal and individual differences in color preferences. Color preferences are just one example of how the mind integrates color-entity associations with contextual cues to form judgments about the world, and this study suggests that to understand these effects and preferences more generally, it is important to consider temporal and individual differences.

## Notes

1 We recruited as many participants as we could prior to the end of the first week of testing. We did not have a basis for anticipating the attrition rate because we did not know of prior studies conducted with our population that asked participants to come to the lab each week for short testing sessions (approximately 5 minutes) over the course of the semester.

2 Randomizing the order of color presentation and using a continuous line-mark sliding scale helped mitigate the potential for participants to memorize their responses to each color over multiple testing sessions. However, if there were memory effects, they would have likely resulted in similar color preferences over testing session rather than the selective changes in preferences for Leaf Colors but not Non-Leaf Colors that we observed here.

3 The significant correlation between preferences for Leaf Colors and fall entities was not significantly greater than the non-significant correlation between preferences for Non-Leaf Colors and fall entities (*z* = .83, *p* = .41), preferences for Leaf Colors and winter entities (*z* = 0.96, *p* = .34), or preferences for Non-Leaf Colors and winter-associated entities (z = 1.25, *p* = .21). However, it is meaningful that there was a significant correlation for the one case where the differential activation hypothesis predicted it would be significant (Leaf Colors with fall entities) and no significant correlations in the other three cases where the hypothesis did not predict a relation (Non-Leaf Colors with fall entities, Leaf Colors with winter entities, and Non-Leaf Colors with winter entities).

## Appendix

**Table A1.** CIE 1931 xyY Coordinates and Munsell Coordinates for the 32 Chromatic Colors (Palmer & Schloss, 2010) and CIE 1931 Values for the Five Achromatic Colors (CIE Illuminant C) (Schloss et al., 2013).

Color	x	y	Y	Hue	Value/Chroma
Red					
Saturated	0.549	0.313	22.93	5 R	5/15
Light	0.407	0.326	49.95	5 R	7/8
Muted	0.441	0.324	22.93	5 R	5/8
Dark	0.506	0.311	7.60	5 R	3/8
Orange					
Saturated	0.513	0.412	49.95	5 YR	7/13
Light	0.399	0.366	68.56	5 YR	8/6
Muted	0.423	0.375	34.86	5 YR	6/6
Dark	0.481	0.388	10.76	5 YR	3.5/6
Yellow					
Saturated	0.446	0.472	91.25	5 Y	9/12
Light	0.391	0.413	91.25	5 Y	9/6.5
Muted	0.407	0.426	49.95	5 Y	7/6.5
Dark	0.437	0.450	18.43	5 Y	5/6.5
Chartreuse					
Saturated	0.387	0.504	68.56	5 GY	8/11
Light	0.357	0.420	79.90	5 GY	8.5/6
Muted	0.360	0.436	42.40	5 GY	6.5/6
Dark	0.369	0.473	18.43	5 GY	4.5/6
Green					
Saturated	0.254	0.449	42.40	3.75 G	6.5/11.5
Light	0.288	0.381	63.90	3.75 G	7.75/6.25
Muted	0.281	0.392	34.86	3.75 G	6/6.25
Dark	0.261	0.419	12.34	3.75 G	3.75/6.25
Cyan					
Saturated	0.226	0.335	49.95	5 BG	7/9
Light	0.267	0.330	68.56	5 BG	8/5
Muted	0.254	0.328	34.86	5 BG	6/5
Dark	0.233	0.324	13.92	5 BG	4/5
Blue					
Saturated	0.200	0.230	34.86	10 B	6/10
Light	0.255	0.278	59.25	10 B	7.5/5.5
Muted	0.241	0.265	28.90	10 B	5.5/5.5
Dark	0.212	0.236	10.76	10 B	3.5/5.5
Purple					
Saturated	0.272	0.156	18.43	5 P	4.5/17
Light	0.290	0.242	49.95	5 P	7/9
Muted	0.287	0.222	22.93	5 P	5/9
Dark	0.280	0.181	7.60	5 P	3/9
Achromatic					
Black	0.310	0.316	0.30		
Dark gray	0.310	0.316	12.34		
Med Gray	0.310	0.316	31.88		
Light Gray	0.310	0.316	63.90		
White	0.310	0.316	116.00

**Table A2.** Objects, Entities and Events That Were Associated with Fall Entities.

Apple bobbing	Football	Pumpkin pie
Apple cider	Gourds	Pumpkin spiced ales and food
Apples	Halloween	Pumpkin spiced latte
Baskets full of apples	Halloween costumes	Pumpkin treats
Blowing leaves	Halloween decorations	Pumpkins
Changing leaves	Harvest festival	Raking/picking up leaves
College football starts	Hayrides	Scarecrows
Colorful leaves	Leaf-watching	Shorter daylight or longer nights
Cooler weather	Leaves	Thanksgiving
Corn	Lots of decorations with hay	Trick or treat
Corn mazes	NFL	Turkey
Eating Thanksgiving dinner	October fest	
Fall decorations	People cutting firewood	
Fall equinox	Piles of leaves	
Falling leaves	Pumpkin carving	
Farm stands selling pumpkins, gourds, fall flowers like mums	Pumpkin patches	

*Note.* From data collected and reported in Schloss et al. (2017)

**Table A3.** Objects, Entities, and Events That Were Associated with Winter.

Bare trees	Freezing temperatures	Seeing breath
Being indoors	Gloves	Shorter daylight and longer nights
Birds flying south	Hanukkah	Shoveling snow
Bitter cold	Harsh and chilly winds	Sipping hot chocolate under covers and watching a movie
Blizzards	Heavy coats	Skiing
Boots	Hockey	Sledding
Building a snowman	Holiday music	Sleet
Bundles of wood	Holiday shopping	Slick sidewalks
Bundling up	Holidays	Slippery roads
Christmas	Hoodies	Snow
Christmas cookies	Hot chocolate	Snow angels in the yard
Christmas lawn decorations	Ice	Snow blowers
Christmas lights	Ice covering things	Snow storms
Christmas lights on in houses or buildings	Ice fishing	Snowball fights
Christmas music	Ice on trees	Snowboarding
Christmas music on radio	Ice skating	Snowfall
Christmas parties	Icicles	Snowmen
Christmas presents	Making snowballs	Snowmobile
Christmas sweaters	Mittens	Snowplows
Christmas tree	New Year’s celebration	Staying indoors
Christmas or holiday decorations	New Year’s Day	Stockings
Cinnamon tea	New Year’s drinking	Sweaters
Coats or jackets	New Year’s Eve	Toboggans
Cold weather	No sun	Trees covered in snow
Compact and cold ground	People cutting firewood	Turning up the heat
Cooler weather	Peppermint	TV specials
Cuddling by the fireplace or heater	Peppermint chocolate cocoa	Wearing more clothing
Dark days	Playing in snow	Winter break
Ear muffs	Presents	Winter gear
Eggnog	Santa Claus	Winter solstice
Evergreens	Scarves	Winter squash
Fire in fireplace	Secret Santa	Wool sweaters

*Note.* From data collected and reported in Schloss et al. (2017).
